# Modeling and analysis of hybrid-blood nanofluid flow in stenotic artery

**DOI:** 10.1038/s41598-024-55621-5

**Published:** 2024-03-05

**Authors:** Lubna Sarwar, Azad Hussain, Muhammad Bilal Riaz, Sobia Akbar

**Affiliations:** 1 Department of Mathematics, University of Gujrat, Gujrat, 50700 Pakistan; 2 IT4Innovations, VSB – Technical University of Ostrava, Ostrava, Czech Republic; 3 Department of Computer Science and Mathematics, Lebanese American University, Byblos, Lebanon

**Keywords:** Arterial stenosis, Newtonian fluid, Numerical solution, Bio-nanofluid, Hybrid NPs, Mathematics and computing, Applied mathematics

## Abstract

Current communication deals with the flow impact of blood inside cosine shape stenotic artery. The under consideration blood flow is treated as Newtonian fluid and flow is assumed to be two dimensional. The governing equation are modelled and solved by adopting similarity transformation under the stenosis assumptions. The important quantities like Prandtl number, flow parameter, blood flow rate and skin friction are attained to analyze the blood flow phenomena in stenosis. The variations of different parameters have been shown graphically. It is of interest to note that velocity increases due to change in flow parameter gamma and temperature of blood decreases by increasing nanoparticles volume fraction and Prandtl number. In the area of medicine, the most interesting nanotechnology approach is the nanoparticles applications in chemotherapy. This study provides further motivation to include more convincing consequences in the present model to represent the blood rheology.

## Introduction

The cardiovascular component of circulatory system is made up of blood, blood vessel and heart. By pumping action of heart, blood circulates throughout the body. Sometimes blood vessel supplying blood to the body is narrowed due to plaque or unnatural growth of artery, known as arterial stenosis. Arterial stenosis happens when the aortic valve of heart narrows and doesn’t fully open, which reduces blood flow from heart to the main artery. Stenotic arteries can cause complication including blood clots, stroke, heart failure and arrhythmias. It would be better to have a clear idea of basics of fluid mechanics to acknowledge the mechanics of blood circulation. R. Ellahi et al.^1^ discussed the behavior of flow of Prandtl blood in stenosis by considering the porous walls of arteries and to obtain solution the perturbation method is used and analyzed the effects of various parameters like slip parameter, stenosis height, stress component, magnetic parameter and Darcy’s number. Experimental and computational efforts have been considered by various researchers^2–6^ to acknowledge the mechanics of blood flow through stenosis.

The applied nanoparticles in nanofluid are made of oxides (Al_2_O), carbides (SiC), metals (Cu, Al), nitrides (SiN, AiN) or non-metals (graphite, carbon nanotubes). Composition of two or more nanoparticles in base fluid is known as hybrid. The base fluid is a conductive fluid, such as ethylene glycol, water, blood other biofluids, lubricants and polymer solutions. Ijaz, S., & Nadeem, S.^7^ investigated blood flow problem through composite stenotic artery by considering porous walls and determine the nanoparticles effects as a medicine. S. R. Shah & R. Kumar^8^ presented the computational analysis of flow problem of blood through constricted artery. Ellahi, R., et al.^9^ described the problem of flow of blood mathematically with the assumption of blood as non-Newtonian fluid. Numbers of research paid attention to study the effects of hybrid nanoparticles^10–13^. Ali, A., et al.^14^ analyzed the behavior of hybrid nanoparticles with addition of magnetohydrodynamic effects. Ali, A., et al.^15^ presented the thermal behavior of hybrid nanoparticles with viscous dissipation and heat flux. N. S. Elgazery^16^ presented numerical solution of non-Newtonian hybrid fluid flow with magnetic effects through permeable medium. A. Ahmed & S. Nadeem^17^ discussed various kinds of nanoparticles under the mild stenosis assumptions. They obtained exact solution by adopting Euler Cauchy method and presented their results for velocity and temperature through graphs and tables. Kh. S. Mekheimer et al.^18^ resulted that nanoparticles can cure cancer because these particles have high atomic number which produce heat and solved governing equations with nanoparticles and thermal energy by using long wavelength assumption.

Present communication has been undertaken by motivated above interesting facts. The unique aspect of delving into blood flow issues within stenosis lies in the application of advanced mathematical modeling. This approach enables a comprehensive examination of the intricate physiological processes involved, utilizing mathematical equations to quantify the effects of stenosis on blood flow. For future research, there is an opportunity to enhance existing mathematical models by incorporating more realistic parameters. This could involve considering patient specific characteristics, accounting for dynamic changes in stenosis severity over time, or incorporating variations in blood viscosity. The practical insights gained from improved mathematical models and simulations offer a promising avenue for addressing the complexities of blood flow in stenotic conditions, making the research appealing to readers interested in both theoretical advancements and practical applications in the field. We analyzed the flow of blood in the narrow artery with addition of nanoparticles by considering non-Newtonian nature of blood. The obtained PDE’s are transformed into ODE’s with the use of similarity transformations. Numerical solution has been calculated for temperature and velocity of blood by using MATLAB bvp4c. Obtained results are shown graphically and also in tabular form. This innovative approach not only contributes to deeper scientific understanding of blood flow issues associated with stenosis but also paves the way for developing more precise medical interventions and personalized treatment strategies.

## Flow geometry and coordinate system

The following relevant assumptions are made:

i. We considered the flow of blood through stenotic artery of cosine shape constriction.
ii. Blood acts like steady, two-dimensional, incompressible Newtonian fluid.
iii. The length of stenosis is $\frac{{L}_{0}}{2},$ width of unblocked region ${2R}_{0}$, radius of the artery is $R\left(x\right)$ and the maximum values of height is represented by λ.
iv. Blood flow along $x-axis$ and $r-axis$ is perpendicular to the flow.
v. The region of stenosis is chosen as
  1 $$R\left( x \right) = \begin{array}{*{20}c} {R_{0} - \frac{{\uplambda }}{2}\left( {1 + Cos\left( {\frac{4\pi x}{{L_{0} }}} \right)} \right),} & { - \frac{{L_{0} }}{4} < x < { }\frac{{L_{0} }}{4}} \\ = {R_{0} } & {Otherwise} \\ \end{array} ,$$

## Problem formulation and method of solution

The governing steady boundary layer equations of motion, momentum and energy for Newtonian hybrid nanofluid are defined respectively:

2 $$\frac{\partial (ru)}{\partial x}+\frac{\partial (rv)}{\partial r}=0,$$

3 $$\left(u\frac{\partial }{\partial x}+v\frac{\partial }{\partial r}\right)u=\frac{{\upmu }_{hnf}}{{\uprho }_{hnf}}\frac{\partial }{r\partial r}\left(r\frac{\partial u}{\partial r}\right),$$

4 $$\left(u\frac{\partial }{\partial x}+v\frac{\partial }{\partial r}\right)T=\frac{{{\text{k}}}_{hnf}}{{\left(\uprho {C}_{p}\right)}_{hnf}}\frac{\partial }{r\partial r}\left(r\frac{\partial T}{\partial r}\right),$$

Boundary conditions can be specified as:

5 $$\left.\begin{array}{c}u=0, v=0 and T={T}_{1} at r=R(x),\\ \frac{\partial u}{\partial r}=0, \frac{\partial T}{\partial r}=0 at r=0.\end{array}\right\},$$

Physical properties of nanofluids are defined as follows^13^:

6 $$\left.\begin{array}{c}{\rho }_{nf}={\rho }_{f}\left(\left(1-\phi \right)+\phi \frac{{\rho }_{s}}{{\rho }_{f}}\right),\\ {\mu }_{nf}=\frac{{\mu }_{f}}{{\left(1-\phi \right)}^{2.5}},\\ {(\rho {C}_{p})}_{nf}={(\rho {C}_{p})}_{f}\left(\left(1-\phi \right)+\phi \frac{{(\rho {C}_{p})}_{s}}{{(\rho {C}_{p})}_{f}}\right),\\ \frac{{k}_{nf}}{{k}_{f}}=\frac{{k}_{s}+2{k}_{bf}-2\phi \left({k}_{bf}-{k}_{s}\right)}{{k}_{s}+2{k}_{bf}+\phi \left({k}_{bf}-{k}_{s}\right)}.\end{array}\right\}$$

7 $$\left.\begin{array}{c}{\rho }_{hnf}= \left(1-{\phi }_{2}\right)\left(\left(1-{\phi }_{1}\right){\rho }_{f}+{\phi }_{1}{\rho }_{{s}_{1}}\right)+{\phi }_{2}{\rho }_{{s}_{2}},\\ {\mu }_{hnf}=\frac{{\mu }_{f}}{{\left(1-{\phi }_{1}\right)}^{2.5}{\left(1-{\phi }_{2}\right)}^{2.5}},\\ {(\rho {C}_{p})}_{hnf}=\left(1-{\phi }_{2}\right)\left(\left(1-{\phi }_{1}\right){(\rho {C}_{p})}_{f}+{\phi }_{1}{(\rho {C}_{p})}_{{s}_{1}}\right)+{\phi }_{2}{(\rho {C}_{p})}_{{s}_{2}},\\ \frac{{k}_{hnf}}{{k}_{f}}=\frac{{k}_{{s}_{1}}+2{k}_{f}-2{\phi }_{1}\left({k}_{f}-{k}_{{s}_{1}}\right)}{{k}_{{s}_{1}}+2{k}_{f}+{\phi }_{1}\left({k}_{f}-{k}_{{s}_{1}}\right)}\times \frac{{k}_{{s}_{2}}+2{k}_{nf}-2{\phi }_{2}\left({k}_{nf}-{k}_{{s}_{2}}\right)}{{k}_{{s}_{2}}+2{k}_{nf}+{\phi }_{2}\left({k}_{nf}-{k}_{{s}_{2}}\right)},\end{array}\right\}$$

where ${\uprho }_{hnf}$, ${\upmu }_{hnf} {k}_{f}$ and ${k}_{hnf}$ are the density, viscosity and thermal conductivity of hybrid nanofluid of Cu-Al_2_O_3_ nanoparticles and blood, the heat capacity of fluid is ${(\uprho {C}_{p})}_{hnf}$ and values of all these properties are defined in Table 1. The value of $\psi$ for $u$ and $v$ is presented in Eq. (8), the continuity Eq. (2) is satisfied.

**Table 1:** Experimental values for (blood) base fluid and (Al_2_O_3_, Cu) hybrid nanoparticles^12^.

Material	Symbol	$\rho (kg/{m}^{3})$	${C}_{p}(J{kg}^{-1}{K}^{-1})$	$k$(${W{m}^{-1}K}^{-1}$)
Blood	–	1050	3617	0.52
Aluminum oxide	Al_2_O_3_	3970	765	40
Copper	Cu	8933	385	400

8 $$u={r}^{-1}\frac{\partial \psi }{\partial r},$$

Then Eqs. (3), (4) are

9 $$\frac{1}{r}\frac{\partial \psi }{\partial r}\frac{\partial }{\partial x}\left(\frac{1}{r}\frac{\partial \psi }{\partial r}\right)-\frac{1}{r}\frac{\partial \psi }{\partial x}\frac{\partial }{\partial r}\left(\frac{1}{r}\frac{\partial \psi }{\partial x}\right)=\frac{{\upmu }_{hnf}}{{\uprho }_{hnf}}\frac{\partial }{r\partial r}\left(\frac{{\partial }^{2}\psi }{\partial {r}^{2}}-\frac{1}{r}\frac{\partial \psi }{\partial r}\right),$$

10 $$\left( \frac{1}{r}\frac{\partial \psi }{\partial r}\right)\frac{\partial T}{\partial x}-\left(\frac{1}{r}\frac{\partial \psi }{\partial x}\right)\frac{\partial T}{\partial r}=\frac{{{\text{k}}}_{hnf}}{{\left(\uprho {C}_{p}\right)}_{hnf}}\frac{\partial }{r\partial r}\left(r\frac{\partial T}{\partial r}\right).$$

### Similarity analysis

Solution of Eq. (7), (8) is obtained by utilizing the transformation presented in Eq. (11).

11 $$u=\frac{{u}_{0}x}{{L}_{0}}{F}{\prime}\left(\eta \right), v=-\frac{R}{r}\sqrt{\frac{{u}_{0}{\nu }_{f}}{{L}_{0}}}F\left(\eta \right), \eta =\frac{{r}^{2}-{R}^{2}}{2R}\sqrt{\frac{{u}_{0}}{{{\nu }_{f}L}_{0}}} , \theta \left(\eta \right)=\frac{T-{T}_{0}}{{T}_{1}-{T}_{0}},$$

$$\psi =\sqrt{\frac{{u}_{0}{x}^{2}{\nu }_{f}}{{L}_{0}}}RF\left(\eta \right),$$

where $x=\frac{\widetilde{x}}{{L}_{0}}$ and after the successful implementation of useful similarity transformation the equations $(9-10)$ finally becomes:

12 $$\frac{1}{{C}_{1}{C}_{2}}\left[\left(1+2\gamma \eta \right){F}^{{\prime}{\prime}{\prime}}+2\gamma F{\prime}{\prime}\right]+F{F}^{{\prime}{\prime}}-{{F}{\prime}}^{2}=0,$$

where

13 $${C}_{1}={\left(1-{\phi }_{1}\right)}^{2.5}{\left(1-{\phi }_{2}\right)}^{2.5}, {C}_{2}=\left[\left(1-{\phi }_{2}\right)\left\{\left(1-{\phi }_{1}\right)+{\phi }_{1}\frac{{\rho }_{{s}_{1}}}{{\rho }_{f}}\right\}+{\phi }_{2}\frac{{\rho }_{{s}_{2}}}{{\rho }_{f}}\right].$$

14 $$\frac{1}{Pr {C}_{3}}\left[\left(1+2\gamma \eta \right){\theta }^{{\prime}{\prime}}+2\gamma {\theta }{\prime}\right]+F{\theta }{\prime}-{F}{\prime}\theta =0,$$

where

15 $${C}_{3}=\frac{{k}_{hnf}}{{k}_{f}}\left\{\left[\left(1-{\phi }_{2}\right)\left\{\left(1-{\phi }_{1}\right)+{\phi }_{1}\frac{{\left(\rho {c}_{p}\right)}_{{s}_{1}}}{{\left(\rho {c}_{p}\right)}_{f}}\right\}\right]+{\phi }_{2}\frac{{\left(\rho {c}_{p}\right)}_{{s}_{2}}}{{\left(\rho {c}_{p}\right)}_{f}}\right\}.$$

Dimensionless form of Eq. (1) is

16 $$\begin{array}{ll} f=1-\frac{\epsilon }{2}\left(1+Cos\left(4\pi \widetilde{x}\right)\right), & \quad -\frac{1}{4} < \widetilde{x}< \frac{1}{4} \\ = 1 & \quad Otherwise, \\ \end{array}$$

where $f=\frac{R(x)}{{R}_{0}}$ and $\epsilon =\frac{\uplambda }{{R}_{0}}$ is the non-dimensional measure of stenosis in reference artery.

Boundary conditions in dimensionless form are

$$F\left(0\right)=0, { F}{\prime}\left(0\right)=0, \theta \left(0\right)=1 at \eta =0,$$

17 $${F}^{{\prime}{\prime}}\left(\eta \right)=0, \theta {\prime}\left(\eta \right)=0 at \eta =f.$$

The dimensionless quantities in Eqs. (12) and (13) are flow parameter $\gamma =\sqrt{{\nu }_{f}{L}_{0}/{u}_{0}{R}^{2}}$, Prandtl number $Pr={k}_{f}/{(\mu {C}_{p})}_{f}$ and Cu- Al_2_O_3_ nanoparticles concentration are shown by ${\phi }_{1}$ and ${\phi }_{2}$.

### Physical quantities

The physical quantities of flow field i.e., coefficient of Skin friction ${C}_{f}$ and heat transfer $N{u}_{x}$ are described as:

18 $${C}_{f}=\frac{{\tau }_{w}}{\frac{1}{2}{\rho }_{f}{U}_{w}^{2}},$$

19 $$N{u}_{x}=\frac{x{q}_{w}}{{k}_{f}({T}_{w}-{T}_{\infty })},$$

Expression for shear stress ${\tau }_{w}$ and heat flux ${q}_{w}$ can be find as

20 $${\tau }_{w}={\mu }_{hnf}{\left.\frac{\partial u}{\partial r}\right|}_{r=R},$$

21 $${q}_{w}={-k}_{hnf}{\left.\frac{\partial T}{\partial r}\right|}_{r=R}$$

Non-dimensional form of Eqs. (18), (19) becomes

22 $$R{e}_{x}^{1/2}{C}_{f}=\frac{1}{{\left(1-{\phi }_{1}\right)}^{2.5}{\left(1-{\phi }_{2}\right)}^{2.5}}{F}^{{\prime}{\prime}}\left(0\right),$$

23 $$, R{e}_{x}^{-1/2}N{u}_{x}=-\frac{{k}_{hnf}}{{k}_{f}}{\theta }{\prime}\left(0\right),$$

where $R{e}_{x}^{-1/2}$ shows the Reynolds number.

### Numerical solution

Numerical solution of Eqs. (12) and (14) is obtained by using MATLAB bvp4c technique. MATLAB bcp4c solve the boundary values problems for ordinary differential equations. The results for temperature and velocity profiles are obtained and presented graphically.

## Graphical results and explanation

**Figure 1:**
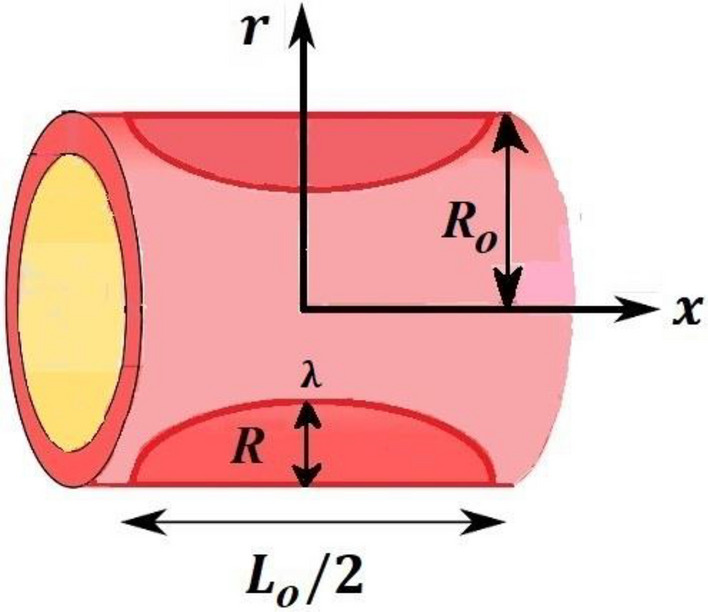
Model of stenotic artery.

**Figure 2:**
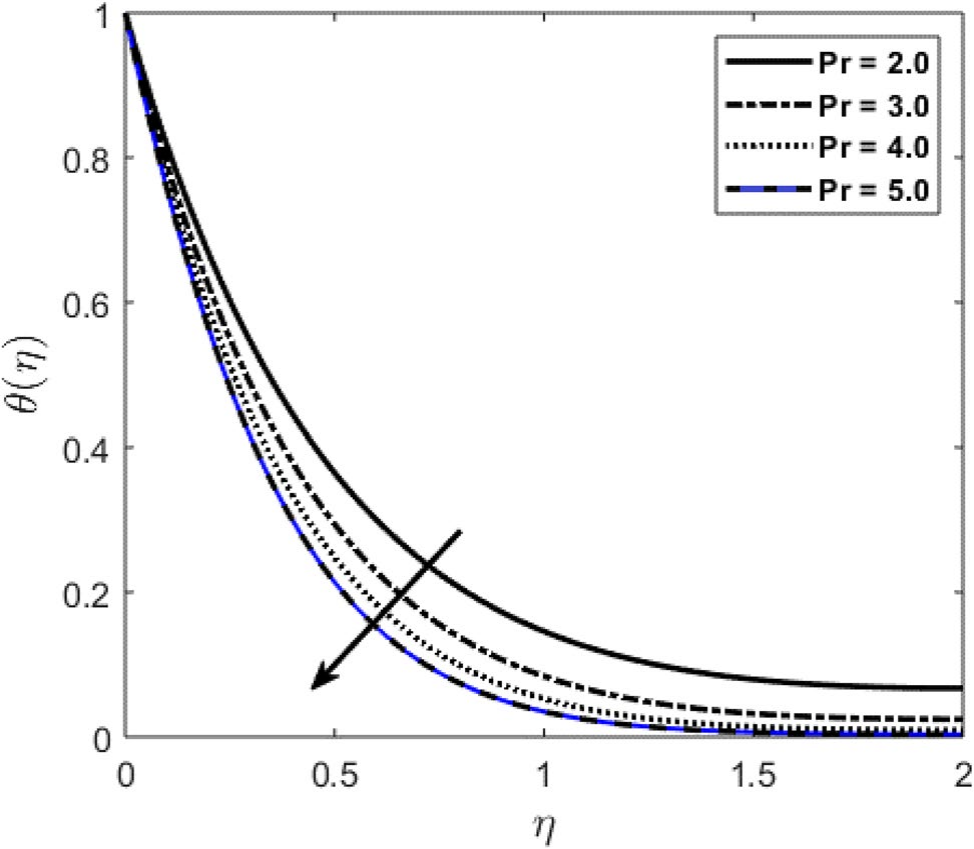
Consequences of $Pr$ on $\theta (\upeta$).

**Figure 3:**
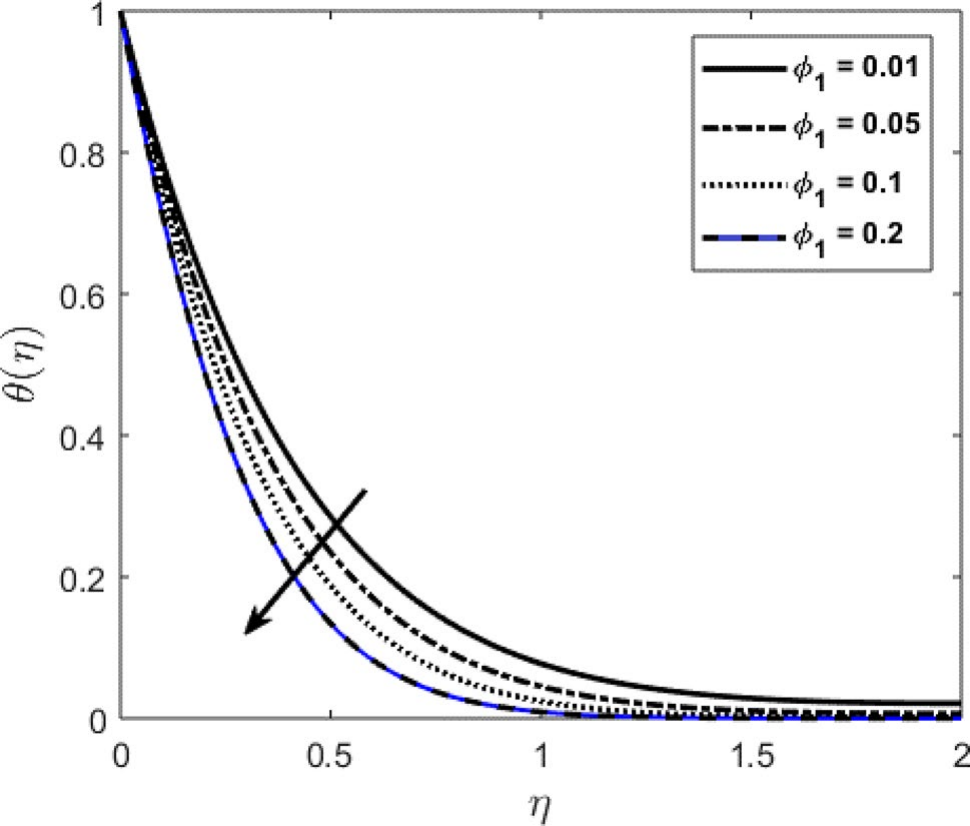
Impact of nanoparticles volume fraction on $\theta (\upeta$).

**Figure 4:**
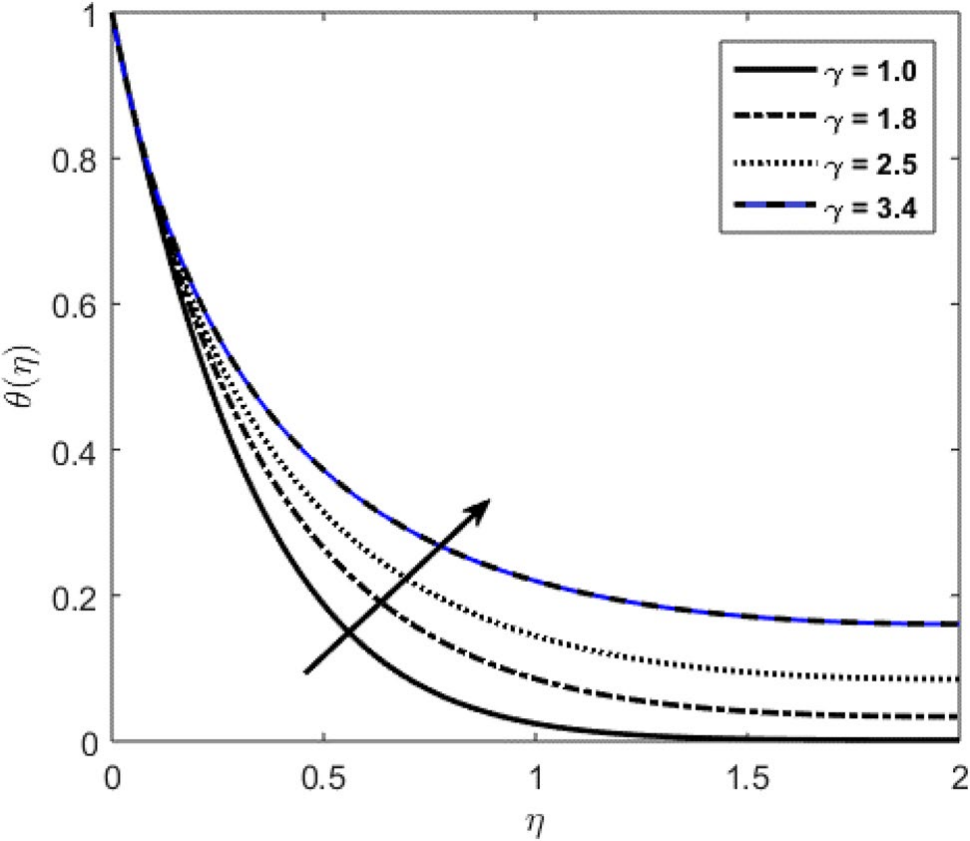
Profile against flow parameter $(\gamma )$ on $\theta (\upeta$).

**Figure 5:**
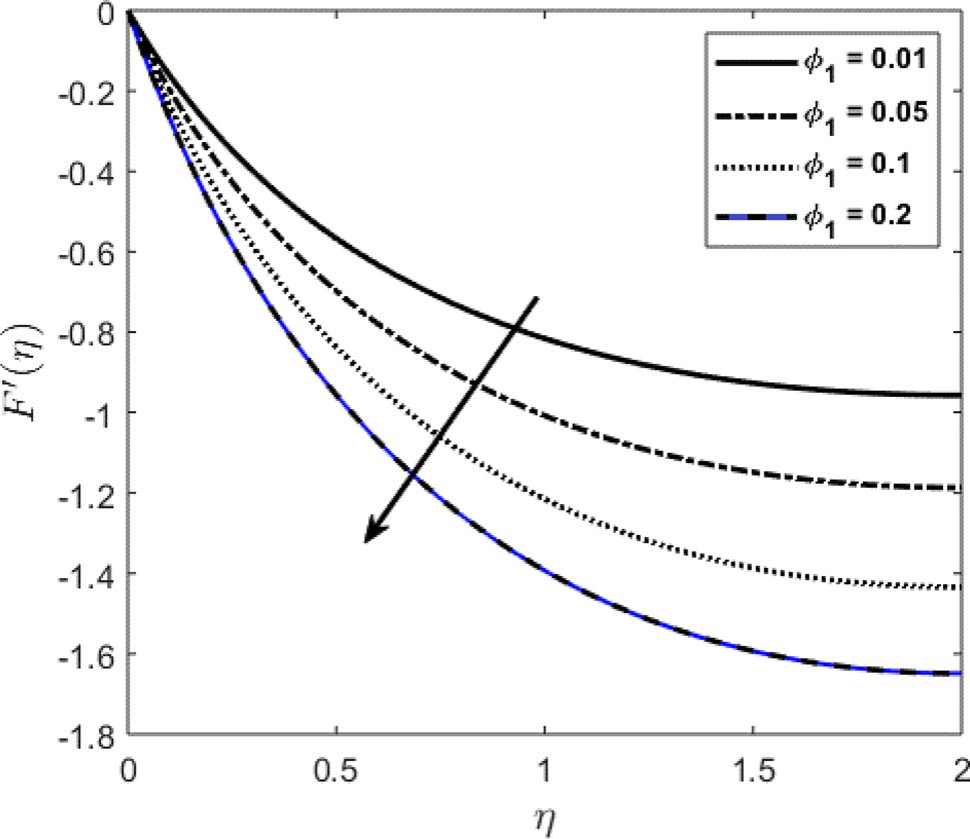
Profile against nanoparticles volume fraction on $F{\prime}(\upeta$).

**Figure 6:**
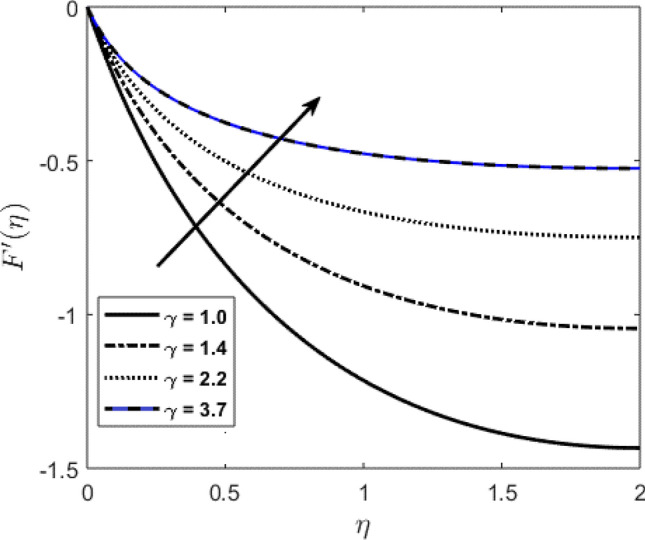
Profile against flow parameter $(\gamma )$ on $F{\prime}(\upeta$).

**Figure 7:**
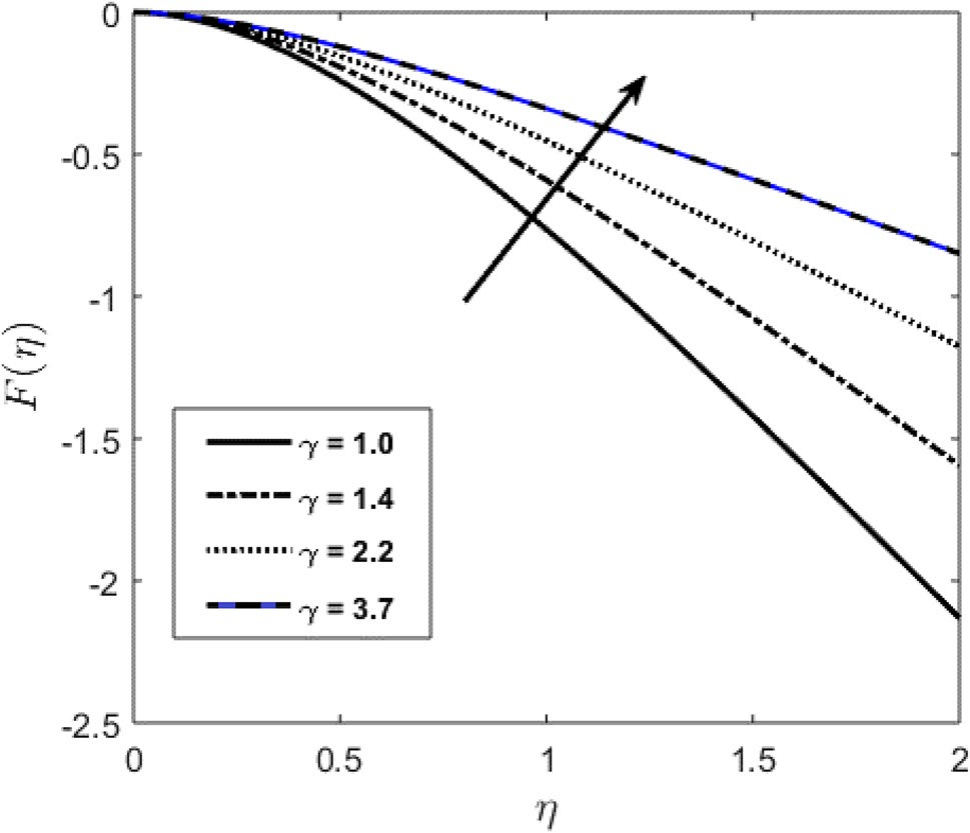
Profile against flow parameter $(\gamma )$ on $F(\upeta$).

**Figure 8:**
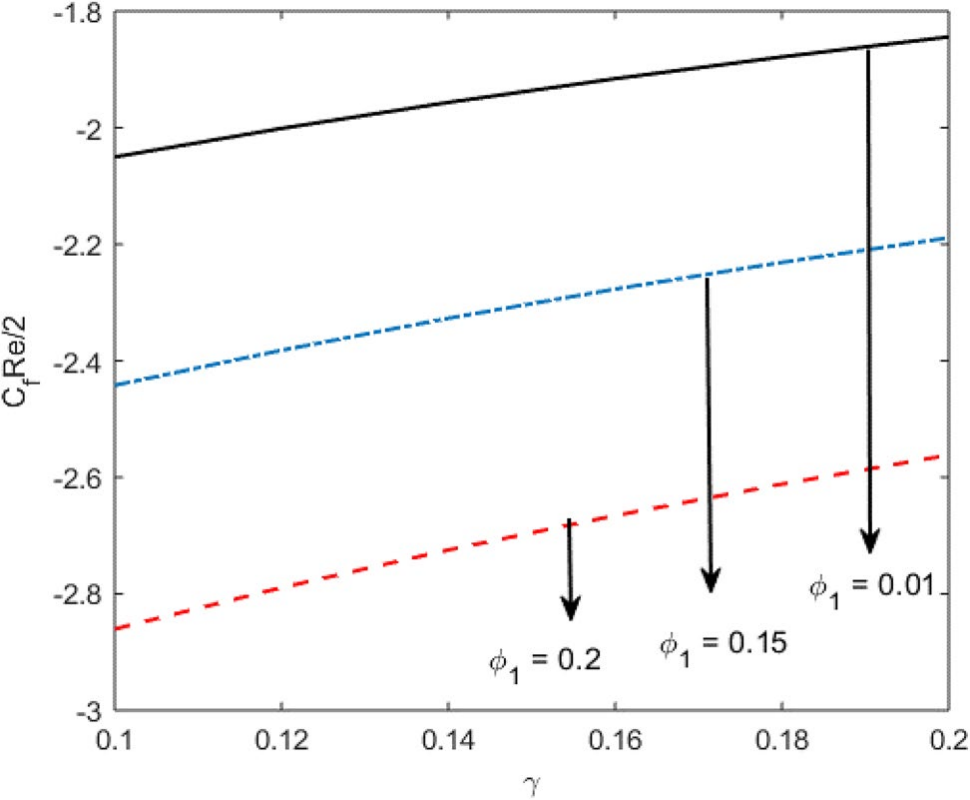
Variations of nanoparticle volume fraction and $\gamma$ on Skin friction coefficient.

**Figure 9:**
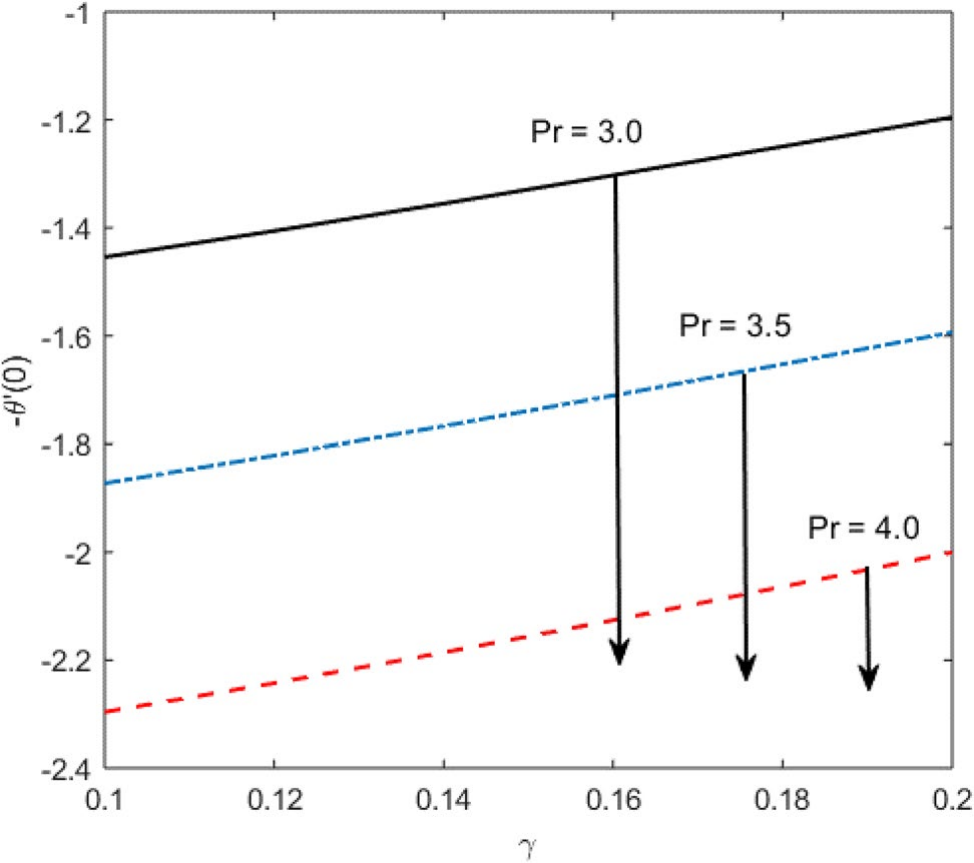
Consequences of $Pr$ and $\gamma$ on heat transfer coefficient.

Blood flow problem through stenotic artery with addition of hybrid nanoparticles is studied. Results of various parameters on stenotic artery are investigated. Figure 1 shows the geometry of stenotic artery. Figure 2 describes the consequences of temperature for $Pr$. Graphical results shows that by rising the $Pr=2.0, 3.0, 4.0, 5.0$ temperature decreases. Basically Pr is a ratio of diffusivity of momentum to thermal. This implies that there is a inverse relation to heat transfer from the wall of artery, for smaller value of Pr the heat diffusion is greater than momentum. Figure 3 depicts the impact of nanoparticles on temperature field and the curve decreases by increasing $\phi =0.01, 0.05, 0.1, 0.2$. Figure 4 depicts the consequences of $\gamma$ on blood temperature and the curve shows increasing behavior by increasing the value of $\gamma =0.1, 1.8, 2.5, 3.4$. Impact of nanoparticles on velocity distribution is shown in Fig. 5. By increasing nanoparticles volume fraction values, velocity curve of blood bending down. This is acceptable with the physical presentation that when the nanoparticles volume fraction rises then due to nanoparticles stacked up in blood, the flow velocity decreases. Figure 6 draws the results of $\gamma$ on velocity profile $F{\prime}(\upeta$). Velocity of blood increases by increasing $\gamma =1.0, 1.4, 2.2, 3$.7. Figure 7 shows the results of $\gamma$ on velocity profile $F(\upeta$). Velocity of blood increases by increasing $\gamma =1.0, 1.4, 2.2, 3$.7. Figure 8 presents skin friction variations due to change in nanoparticles volume fraction and flow parameter. Skin friction profile goes down by rising $\gamma$ values. Figure 9 presents consequences of heat transfer coefficient and the curve shows decreasing behavior. Table 1 describes the values for (blood) base fluid and solid hybrid nanoparticles. Table 2 describes the impact of $Pr$ and $\gamma$ on Nusselt number. Results exhibits that for $Pr$ values coefficient of heat transfer rises by increase in $\gamma$ while goes down. Table 3 shown the results of $\gamma$ and $\phi$ on skin friction. We can conclude that by rising the flow parameter $\gamma$, the values of Skin friction coefficient also rises and by increasing $\phi$, the coefficient of Skin friction decreases. Remarkable properties are show by hybrid nanoparticles which cannot attained in individual state by any component. Mainly the applications of nano drug delivery in biofluid dynamics, for arterial diseases treatments, a detail computational study is presented for hybrid nanoparticles and heat transfer through stenotic artery. Results of present study bay be fruitful during operation procedures in tuning the blood flow.

**Table 2:** Nusselt number variations with respect to $Pr$ and $\gamma$.

$\gamma$	$Pr$	$-\frac{{k}_{nf}}{{k}_{f}}\theta {\prime}(0)$
0.1	3.0	− 1.4544
0.12		− 1.4059
0.14		− 1.3552
0.1		− 1.4544
	3.5	− 1.8724
	4.0	− 2.2959

**Table 3:** Numerical values for skin friction coefficient with respect to $\phi$ and $\gamma .$

$\gamma$	ϕ	$\frac{1}{2}{C}_{f}Re$
0.1	0.01	− 2.0502
0.12		− 2.0012
0.14		− 1.9566
0.1		− 2.0502
	0.15	− 2.4421
	0.2	− 2.8603

## Conclusion

Flow of blood through stenotic artery with addition of hybrid nanoparticles is studied. The mathematical study of blood flow in stenosis involves modeling complex fluid dynamics, vessels geometry and rheological properties to understand the impact of narrowed passages on flow patterns. A numerical method has been used to attain computational solution. Following are the main results of current investigation:

- Increased flow parameter signifies an elevation in the temperature curve.
- The reduction in fluid temperature is associated with the enlargement of nanoparticle size and Prandtl number values.
- Elevating the values of flow parameter enhances blood velocity, whereas introducing variations in nanoparticles leads to a reduction in blood velocity.
- Skin friction curve decreases with an increase in the values of nanoparticle volume fraction $.$
- Nusselt number values vary in relation to $\gamma$ and Pr, influencing the heat transfer characteristics of the system.

## Data Availability

All the data mentioned in this paper is included within the paper.
